# Novel Circular Single-Stranded DNA Viruses among an Asteroid, Echinoid and Holothurian (Phylum: Echinodermata)

**DOI:** 10.1371/journal.pone.0166093

**Published:** 2016-11-17

**Authors:** Elliot W. Jackson, Kalia S. I. Bistolas, Jason B. Button, Ian Hewson

**Affiliations:** Department of Microbiology, Cornell University, Ithaca, New York, United States of America; University of British Columbia, CANADA

## Abstract

Echinoderms are prone to large population fluctuations that can be mediated by pervasive disease events. For the majority of echinoderm disease events the causative pathogen is unknown. Viruses have only recently been explored as potential pathogens using culture-independent techniques though little information currently exists on echinoderm viruses. In this study, ten circular ssDNA viruses were discovered in tissues among an asteroid (*Asterias forbesi)*, an echinoid (*Strongylocentrotus droebachiensis)* and a holothurian (*Parastichopus californicus)* using viral metagenomics. Genome architecture and sequence similarity place these viruses among the rapidly expanding circular *rep*-encoding single stranded (CRESS) DNA viral group. Multiple genomes from the same tissue were no more similar in sequence identity to each other than when compared to other known CRESS DNA viruses. The results from this study are the first to describe a virus from a holothurian and continue to show the ubiquity of these viruses among aquatic invertebrates.

## Introduction

Diseases of echinoderms, particularly echinoids (sea urchins) and asteroids (sea stars), have been extensively documented worldwide and include some of the largest marine epizootics known to date [1–4]. For many of these disease events, a causative pathogen remains undescribed which severely limits the study of the ecology and evolution of infectious diseases of these animals. Of the cases in which a pathogenic agent has been identified or statistically associated with a disease, bacteria and eukaryotic parasites (amoebozoa) are the most well described [5,6]. To date, no fungi have been found associated with echinoderm disease and viruses have only recently been explored as potential pathogens [7]. The discovery of a densovirus linked to a mass morality event of sea stars on the west coast of North America brings to question the role of viruses in other echinoderm diseases [4]. More recently, the discovery of a circular *rep*-encoding single-stranded (CRESS) DNA virus was discovered from tissue of a *Asterias forbesi*, a common sea star on the east coast of North America, exhibiting symptoms similar to the sea star wasting disease observed in the Northeast Pacific. However, no significant correlation was found between viral load/prevalence and symptomatic vs asymptomatic individuals [8]. We sought to further the investigation of CRESS DNA viruses among organisms within the phylum Echinodermata to understand the genotypic divergence and commonality of CRESS DNA viruses among these unique organisms.

Currently the study of echinoderm viruses requires culture-independent methods for the reason that no marine invertebrate cell lines exist. Metagenomics is a powerful tool that can be used to develop molecular diagnostic assays to explore suspect viruses found in animal tissues. However, choosing a virus for further diagnostic assays from the wealth of viral diversity that can be found in the viral metagenomic data can prove to be quite challenging especially since many viruses detected using genomic approaches are highly divergent from cultured representatives. It is important therefore to have a general knowledge of viruses associated with the host of interest when such tools are used for diagnostic purposes as it can guide researchers in identifying potential viral pathogens more confidently. Here we report the findings from viral metagenomic data on a commonality of a viral group, CRESS DNA viruses, among echinoderms. Tissues from three organisms representing three of the five extant classes within the phylum Echinodermata (Asteroidea, Echinoidea and Holothuroidea) were examined for the presence of CRESS DNA viruses, and ten complete CRESS DNA viral genomes were discovered. Multiple CRESS DNA viral genomes were identified from each organism providing a unique opportunity to explore genotypic divergence within and between species. This study reports the first virus to be described from a holothurian and contributes to our knowledge of viruses associated with echinoderms.

## Materials and Methods

Samples were collected from three separate locations spanning a broad geographic range. *Asterias forbesi* were collected from the intertidal from Nahant Bay, Massachusetts, USA (42.4208, -70.9064) in September of 2015 under the Ocean Genome Legacy’s general scientific collection permit (#156386) issued by the Massachusetts Division Marine Fisheries [9]. The *A*. *forbesi* used in the study had symptoms of disease similar to the sea star wasting disease reported on the west coast of the USA. *Strongylocentrotus droebachiensis* were collected from public display tanks at the Vancouver Aquarium, British Columbia, Canada in October 2014. *S*. *droebachiensis* were collected under permit XR 1 2014 issued from the Department of Fisheries and Ocean for Statistical Areas 28 and 29. *Parastichopus californicus* were collected from Ketchikan, Alaska, USA (55.3410, -131.6641) during March of 2015 by the Alaska Department of Fish and Game. The Alaska Department of Fish and Game is the permit issuing authority for non-federally managed species in the State waters so the collection *P*. *californicus* falls under their existing authority. Both *S*. *droebachiensis* and *P*. *californicus* were apparently healthy animals when collected i.e. they had no epidermal lesions or test balding that would be suggestive of disease. However, both *S*. *droebachiensis* and *P*. *californicus* were collected from populations with symptomatic individuals. Viral metagenomic libraries were generated from symptomatic species from these populations though no CRESS-DNA viruses were found among the sequence libraries. Upon collection, all samples were stored at either -20°C or -80°C prior to sample preparation. None of the animals collected for this study involved endangered or protected species.

Viral metagenomic libraries were generated for each specimen per protocols specified in Thurber et al [10]. Samples were sent to the Cornell Institute of Biotechnology for Illumina MiSeq 500 bp sequencing v2 * (e.g. 2 x 250 bp). CLC Genomics Workbench 8.5.1 was used for read quality analysis and assembly. Reads with bases exceeding a quality score of 0.05 or containing ambiguities were discarded. Reads less than 249 nt or greater than 251nt were also discarded. The remaining reads for each library were subjected to *de novo* assembly using CLC Genomics Workbench de Bruijn graph assembler with a minimum contig length of 500. Contigs were annotated by tBLASTx [11] against a curated in-house database of circular ssDNA virus genomes obtained from NCBI with an e-value cutoff 1x10^-5^. Contigs with significant (i.e. e < 1x10^-5^) similarity to circular ssDNA virus genomes were isolated and imported back in CLC Genomics Workbench for read mapping. Reads from the respective libraries were mapped back to contigs using an overlap consensus sequence algorithm with a mis-match cost of 2, insertion cost of 3, deletion cost of 3, length fraction 0.8 and a similarity of 0.5. Contigs were updated based on mapping results. Open reading frames (ORFs) were obtained using CLC Genomic Workbench 8.5.1 by searching both strands for start codons with a minimum codon length of 100. Once the contigs were assessed for these criteria, a BLASTn analysis was conducted to compare the contigs to the closest respective genome to verify the independence of these viral genomes from endogenous viral elements. In addition, contigs were screened for sequence similarity to known laboratory contaminants (BLASTn, e-value 1x10^-5^). Laboratory contaminants were identified through a parallel metavirome preparation containing 0.02um filtered nuclease free water as a template [12].

After the contigs were finalized, the putative *rep* ORFs of each contig was translated using MUSCLE [13] and aligned with other putative *rep* genes from circular ssDNA viruses obtained from NCBI. Using the alignment as a guide, *rep* sequences were manually screened for rolling circle replication (RCR) motifs and SF3 helicase motifs to verify homology to the vRep protein [14]. The translated *rep* sequences were then imported into Sequence Demarcation Tool Version 1.2 (SDTv1.2) [15] for pairwise amino acid similarity analyses to sequences with strong similarity based on BLASTx results against the NCBI non-redundant database (S2 Table). Hydrophobicity of the amino acid sequences of the putative capsid protein were calculated using Kyte and Doolittle [16]. Finally, contigs were screened for the conversed nonanucleotide motif and stem-loop structures were predicted using Mfold [17].

## Results

**Fig 1:**
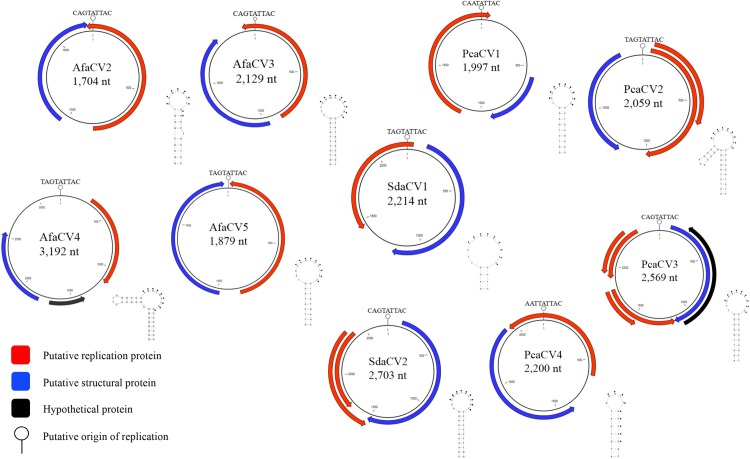
Genome illustrations of ssDNA viruses.

Ten complete circular ssDNA viral genomes were identified from metagenomic analysis of purified DNA from viral particles of tissue from *A*. *forbesi* (n = 4), *S*. *droebachiensis* (n = 2) and *P*. *californicus* (n = 4) (Fig 1). After mapping the reads back to the contigs, the average coverage between the contigs was 572x ranging from 16x to 2,270x (Table 1). The size of the genomes spanned 1,704 to 3,192 nucleotides all encoding at least 2 ORFS exhibiting both unisense and ambisense orientation. All genomes contained a putative *rep* gene that had significant similarity (BLASTx, e-value < 1x10^-5^) to a putative *rep* gene from another circular ssDNA virus genome. The amino acid sequence length of the putative *rep* genes ranged from 214aa to 318aa while the putative *cap* gene ranged from 215aa to 467aa. Nonanucleotide motifs were all found within a predicted stem-loop structure (Fig 1). All stem-loop structures predicted are energetically favorable (ΔG < 0). TAGTATTAC and CAGTATTAC were the most represented nonanucleotide motifs, 4 of 10 each (Table 1). None of the viral genomes contained all conserved residues of motifs found in a vRep protein that would definitively place it into one of the four well defined eukaryotic circular ssDNA viral groups (S1 Table).

**Table 1:** Genome description, coverage and characteristics of ssDNA viruses identified in this study based on genomic features.

Host	Collection Site	Name	Health status upon collection^1^	Genome Coverage	Genome Size (nt)	Putative *rep* (aa)^3^	Putative *cap* (aa)^2^	Nonanucleotide motif	Type	Orientation
*Asterias forbesi* (sea star)	Nahant Bay, Massachusetts	AfaCV2	Symptomatic	16	1,704	293	215	CAGTATTAC	IV	Ambisense
		AfaCV3		64	2,129	316	302*	CAGTATTAC	IV	Ambisense
		AfaCV4		205	3,192	285	245	TAGTATTAC	V	Unisense
		AfaCV5		44	1,879	286	289	TAGTATTAC	IV	Ambisense
*Strongylocentrotus droebachiensis* (sea urchin)	Vancouver, British Columbia	SdaCV1	Asymptomatic	349	2,214	270	356	TAGTATTAC	II	Ambisense
		SdaCV2		380	2,703	275†	467	CAGTATTAC	II	Ambisense
*Parastichopus californicus* (sea cucumber)	Ketchikan, Alaska	PcaCV1	Asymptomatic	273	1,970	307	279	CAATATTAC	V	Unisense
		PcaCV2		2,270	2,059	318†	243	TAGTATTAC	I	Ambisense
		PcaCV3		131	2,569	214†	314*	CAGTATTAC	VI	Unisense
		PcaCV4		1,989	2,200	292	345	AATTATTAC	VI	Unisense

^1^ Organisms labeled as symptomatic exhibited signs of disease (loss of arm, epidermal lesions, loss of turgor).

^2^Non-*rep* encoding ORFs were identified as putative capsid proteins based on BLASTx results against the NCBI non-redundant database. Non-*rep*-encoding ORFs that did not have significant similarity (evalue < 1e^-5^) are denoted (*).

^3^ Genomes containing multiple ORFS encoding the same putative function are marked with (†). The ORF with the lowest evalue from the BLASTx results against the NCBI non-redundant database are represented in this table.

**Fig 2:**
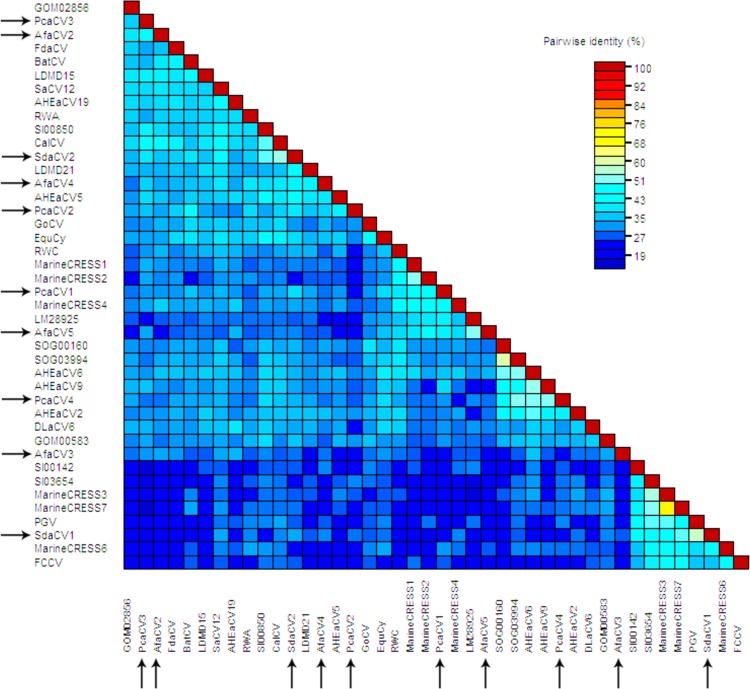
Pairwise amino acid sequence identity analysis of the putative *rep* genes including sequences with strong similarity based on BLASTx results against the NCBI non-redundant database. Arrows indicate genomes obtained in this study.

BLASTx analyses of the *rep* gene of each viral genome show strong homology by similarity to other replication genes of circular ssDNA viruses in the NCBI database. The majority of *rep* genes were most similar to CRESS DNA viral *rep* sequences identified through metagenomic surveys of environmental samples (Fig 2). AfaCV was not identified from the *A*. *forbesi* sample and viral genomes that were found did not show a stronger similarity to AfaCV when compared to other CRESS DNA viruses. Similar to most CRESS DNA viruses discovered through metagenomic analysis, the amino acid percent identities for each of the *rep* genes ranged from 34–57% (Fig 2). 8 of the 10 putative capsid ORFs had significant similarities from the BLASTx analyses to other putative capsid proteins of circular ssDNA viruses. The lack of similarity in 2 of the 10 putative capsid ORFs is not unexpected because the capsid protein is generally found to be less conserved on the amino acid level. In attempt to further verify the annotation of the putative capsid gene, hydrophobicity across the amino acid sequence was investigated. Circovirus capsid proteins are rich in basic amino acids in the N-terminus region which was generally observed along these putative capsid amino acid sequences (S1–S10 Figs). Taken together, the sample preparation and thorough examination of both protein encoding and non-protein encoding genetic elements highly suggest the circular genomes identified in this study are viral and not another intracellular episomal element that replicates via a rolling circle mechanism such as a plasmid or helitron [18].

## Discussion

The use of culture-independent techniques (metagenomics and degenerate PCR assays) has illuminated the widespread nature and diversity of circular *rep*-encoding single-stranded (CRESS) DNA viruses [19,20]. In fact, no two same genotypes of CRESS DNA viruses have been found within or between environments sampled. The evolutionary breadth of eukaryotes that circular ssDNA viruses have now been associated with span the length of animal evolution from Ctenophora to Chordata [21,22]. Aquatic invertebrates, apart from insects, have been the most heavily surveyed and studied eukaryotic organisms for CRESS DNA viruses particularly organisms in the subphylum *Crustacea* notably amphipods [20,23], cladocerans [24], decapods [20] and copepods [25]. CRESS DNA viruses have been found in other aquatic invertebrates including mollusks (phylum Mollusca) [20,26,27] and corals (phylum Cnidaria) [20]. The majority of CRESS DNA viral genotypes however are biased towards arthropods. Identifying novel genotypes from a broader range of host organisms could clarify an evolutionary pattern of eukaryotic circular ssDNA viruses particularly within the family *Circoviridae*. The results from this study show that circular ssDNA viruses are commonly associated with other aquatic animals other than crustaceans and continued viral surveys of marine and freshwater fauna will most likely report similar results. Whether these viruses have the same or different relationships with hosts from disparate groups remains unknown.

The pathogenicity and ecological cost of infection of CRESS DNA viruses currently remains a mystery. The lack of immortal cell lines available for many freshwater and marine organisms severely limits the ability to establish virulence of their associated viruses. Additionally, it is often difficult to implicate these viruses in disease for the reason that these viruses are often associated with asymptomatic hosts. Therefore, correlation based studies have been used to infer the potential cost of infections by these viruses on host populations. The conclusions from these studies are mixed. CRESS DNA viruses have been correlated with mortality rates [24] and stressed host populations [23]. Fahsbender et al reported an association of a CRESS DNA virus with disease symptoms of *A*. *forbesi*, but this correlation was not statistically significant. Nevertheless, a general observation from these correlation-based studies indicate that CRESS DNA viruses are highly prevalent and persistent among host populations [22,24]. These patterns of infection, in addition to the extreme diversity of viral genotypes, suggest that CRESS-DNA viruses are not strongly virulent and cost of infection may be cryptic or even mutualistic. The results from this study contribute to our understanding of the nanobiome of echinoderms and further verify the widespread nature of CRESS DNA viruses among aquatic invertebrates.

## Supporting Information

S1 Fig Hydrophobic plot of hypothetical capsid protein of AfaCV2. (PDF)

S2 Fig Hydrophobic plot of hypothetical capsid protein of AfaCV3. (PDF)

S3 Fig Hydrophobic plot of hypothetical capsid protein of AfaCV4. (PDF)

S4 Fig Hydrophobic plot of hypothetical capsid protein of AfaCV5. (PDF)

S5 Fig Hydrophobic plot of hypothetical capsid protein of SdaCV1. (PDF)

S6 Fig Hydrophobic plot of hypothetical capsid protein of SdaCV2. (PDF)

S7 Fig Hydrophobic plot of hypothetical capsid protein of PcaCV1. (PDF)

S8 Fig Hydrophobic plot of hypothetical capsid protein of PcaCV2. (PDF)

S9 Fig Hydrophobic plot of hypothetical capsid protein of PcaCV3. (PDF)

S10 Fig Hydrophobic plot of hypothetical capsid protein of PcaCV4. (PDF)

S1 Table RCR and SF3 Helicase motifs found in circular ssDNA viruses. Conserved amino acid motifs obtained from alignments of putative *Rep* gene using MUCLE.
(PDF)

S2 Table *Rep* sequences pulled from NCBI for SDT analysis. (PDF)

S3 Table GenBank accession #s for novel virus genomes identified in this study. (PDF)
