# Supplementary figures and images for: Novel Circular Single-Stranded DNA Viruses among an Asteroid, Echinoid and Holothurian (Phylum: Echinodermata)
Source: PLoS One. 2016 Nov 17;11(11):e0166093. doi: 10.1371/journal.pone.0166093 (PMC5113903; doi:10.1371/journal.pone.0166093)

**S1 Fig. Hydrophobic plot of hypothetical capsid protein of AfaCV2.**

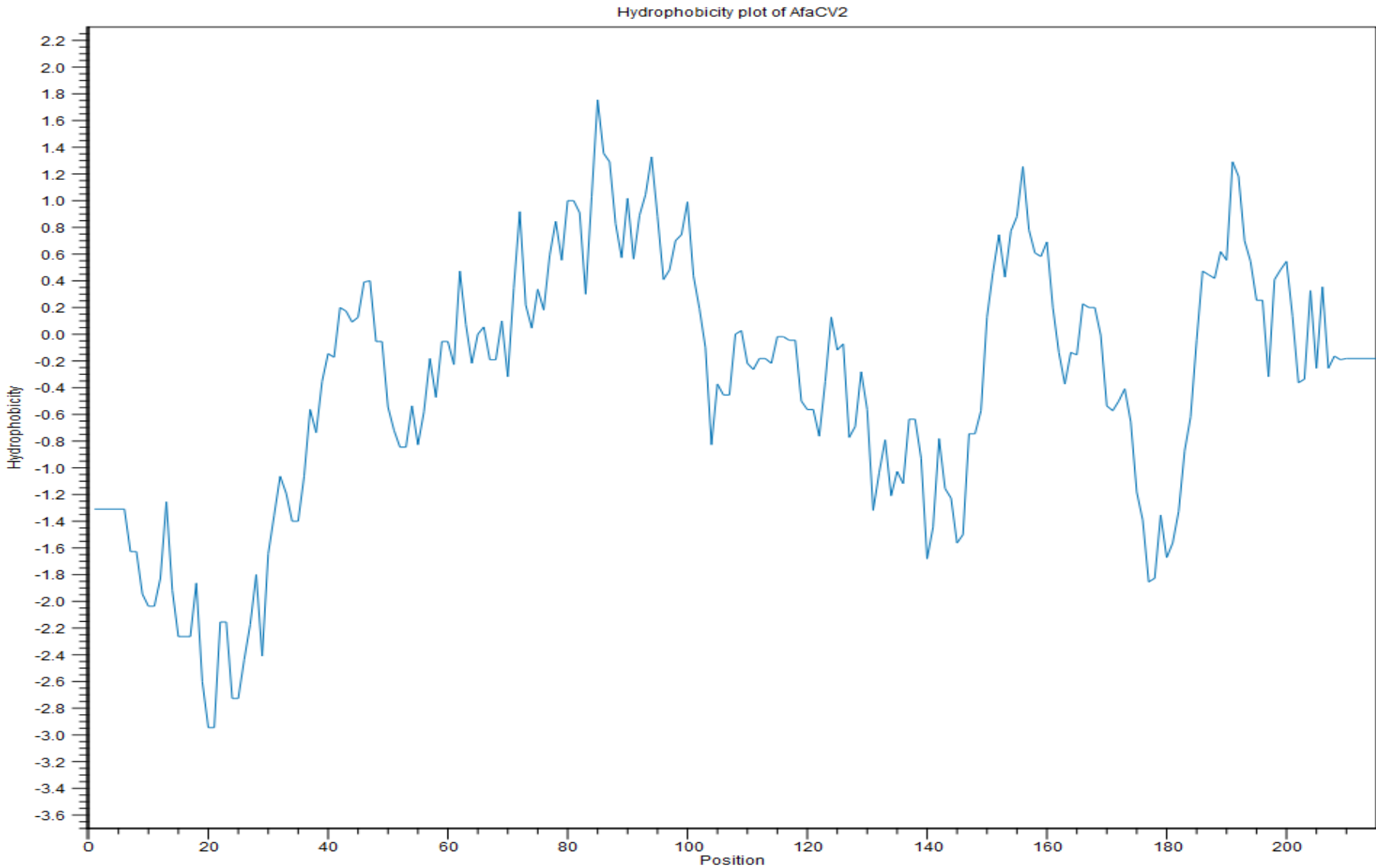

Supplement: S1 Fig — (PDF) [file pone.0166093.s001.pdf]

**S2 Fig. Hydrophobic plot of hypothetical capsid protein of AfaCV3.**

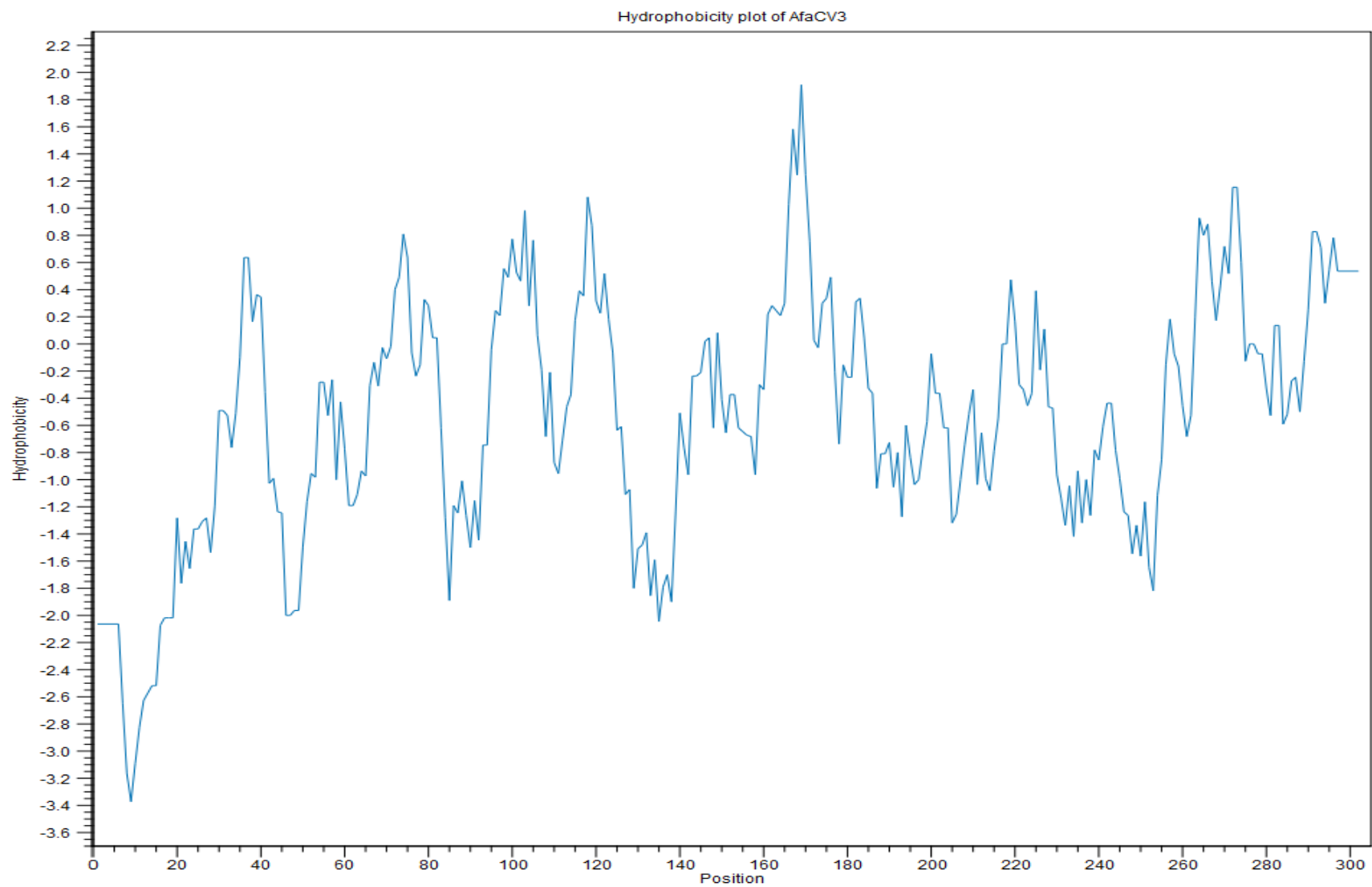

Supplement: S2 Fig — (PDF) [file pone.0166093.s002.pdf]

**S3 Fig. Hydrophobic plot of hypothetical capsid protein of AfaCV4.**

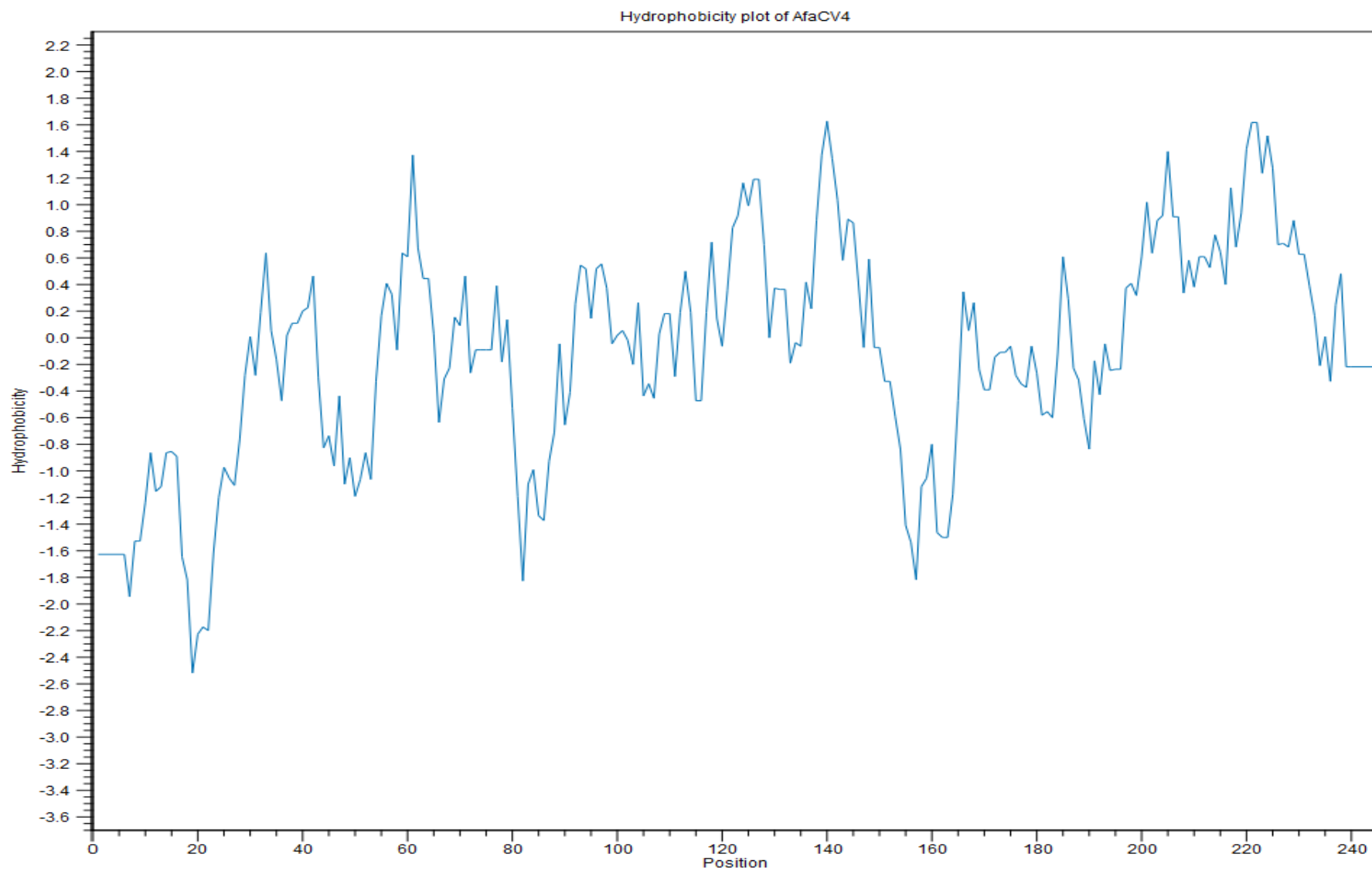

Supplement: S3 Fig — (PDF) [file pone.0166093.s003.pdf]

**S4 Fig. Hydrophobic plot of hypothetical capsid protein of AfaCV5.**

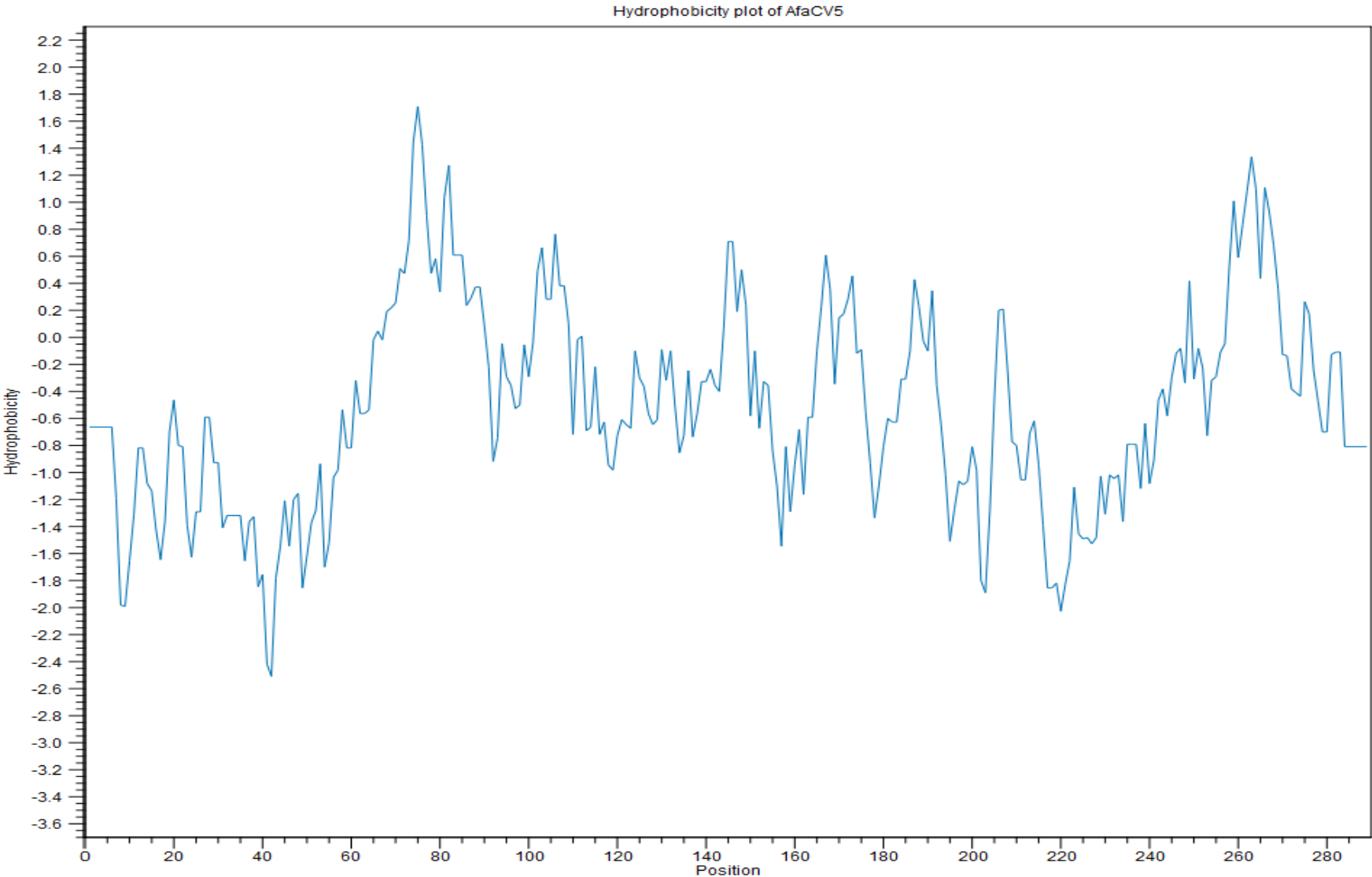

Supplement: S4 Fig — (PDF) [file pone.0166093.s004.pdf]

**S5 Fig. Hydrophobic plot of hypothetical capsid protein of SdaCV1.**

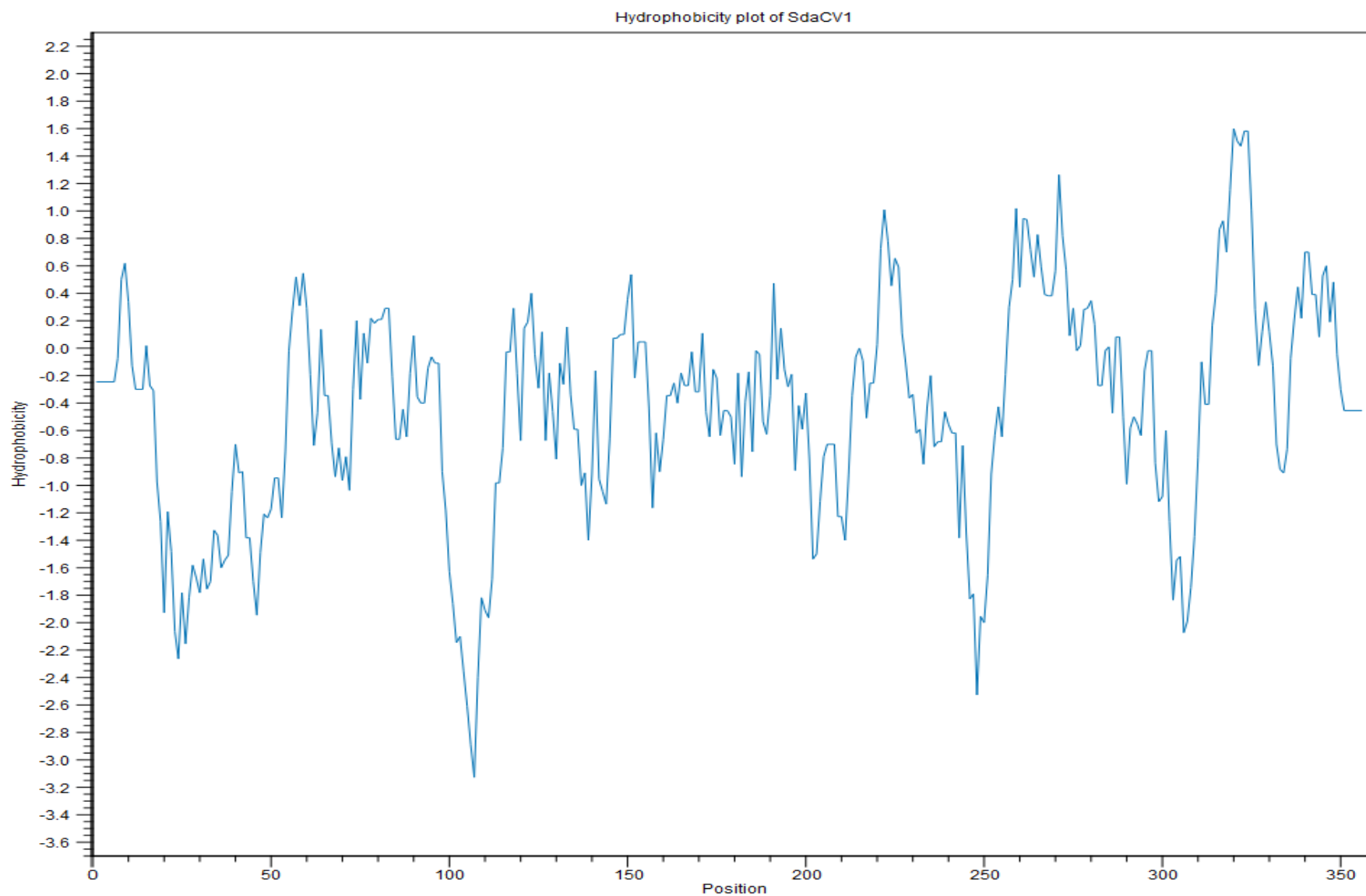

Supplement: S5 Fig — (PDF) [file pone.0166093.s005.pdf]

**S6 Fig. Hydrophobic plot of hypothetical capsid protein of SdaCV2.**

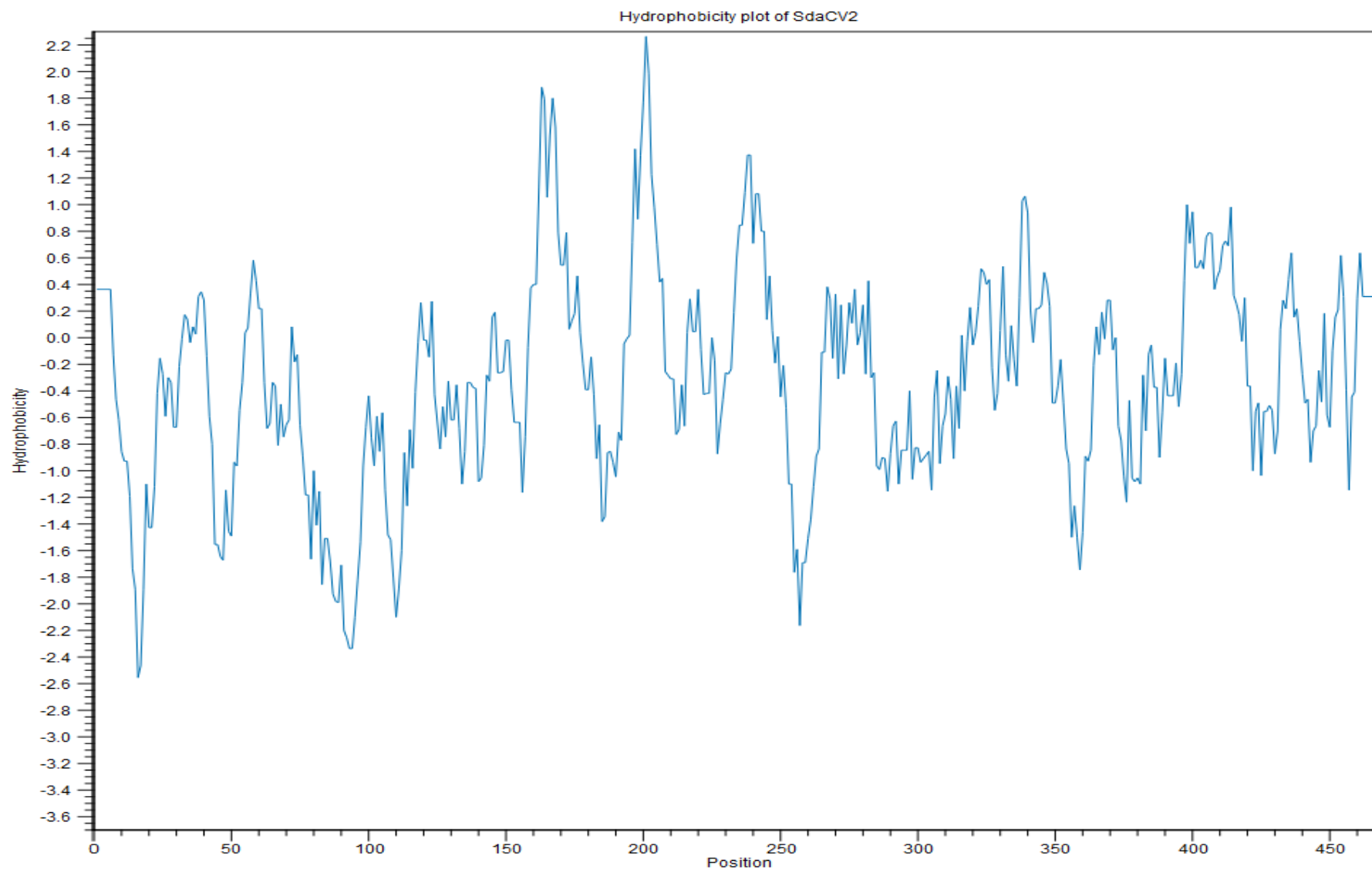

Supplement: S6 Fig — (PDF) [file pone.0166093.s006.pdf]

**S7 Fig. Hydrophobic plot of hypothetical capsid protein of PcaCV1.**

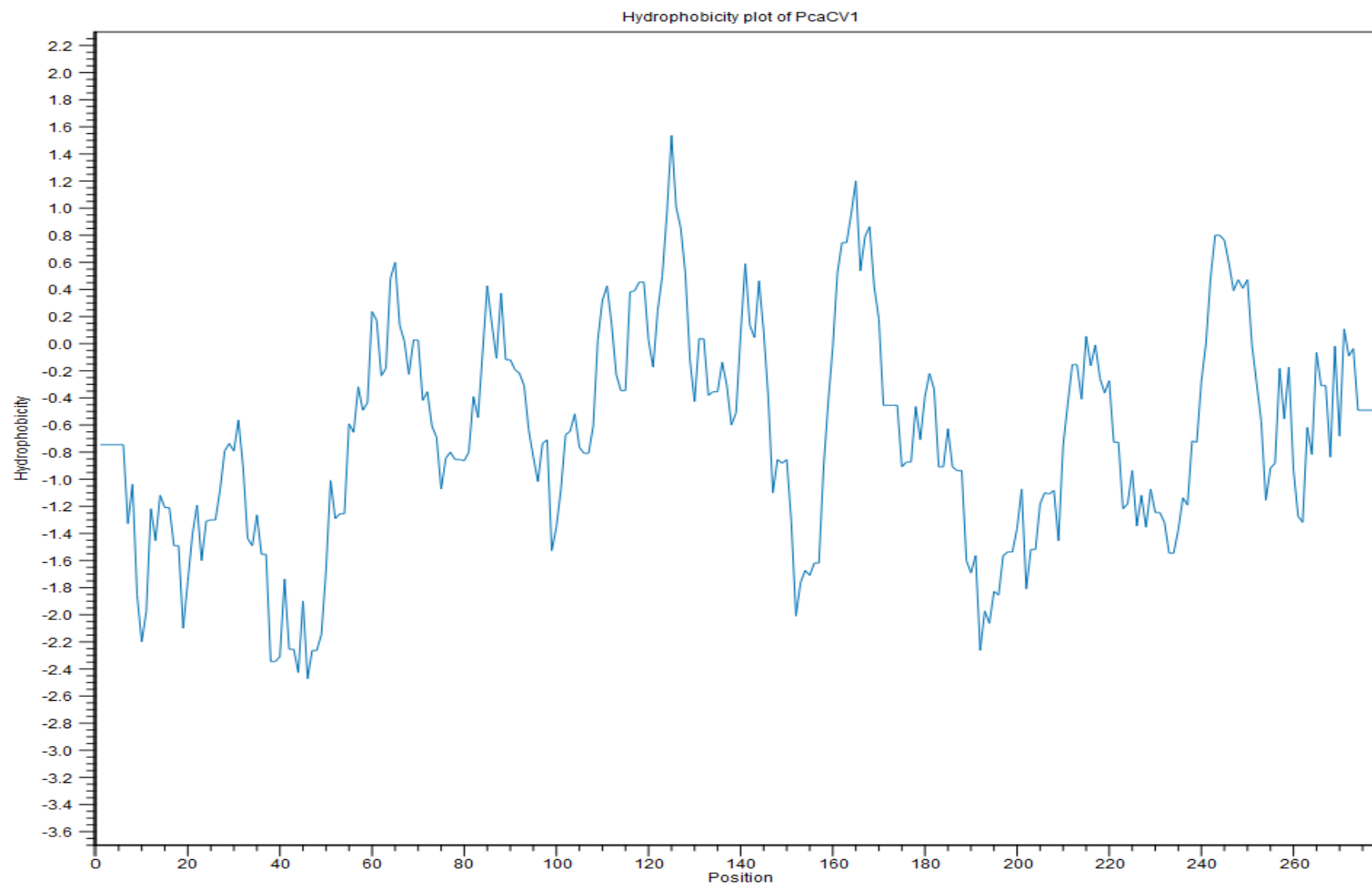

Supplement: S7 Fig — (PDF) [file pone.0166093.s007.pdf]

**S8 Fig. Hydrophobic plot of hypothetical capsid protein of PcaCV2.**

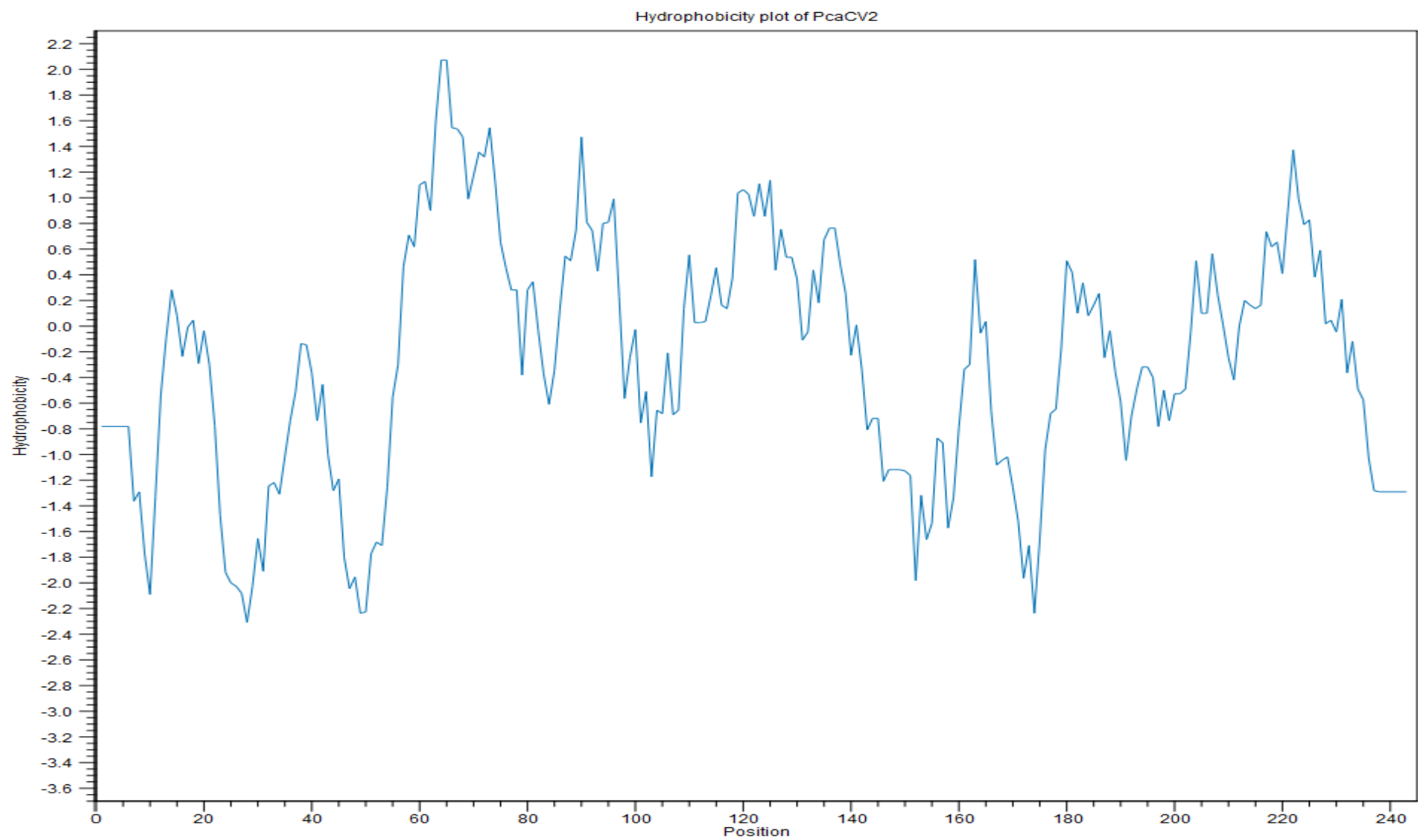

Supplement: S8 Fig — (PDF) [file pone.0166093.s008.pdf]

**S9 Fig. Hydrophobic plot of hypothetical capsid protein of PcaCV3.**

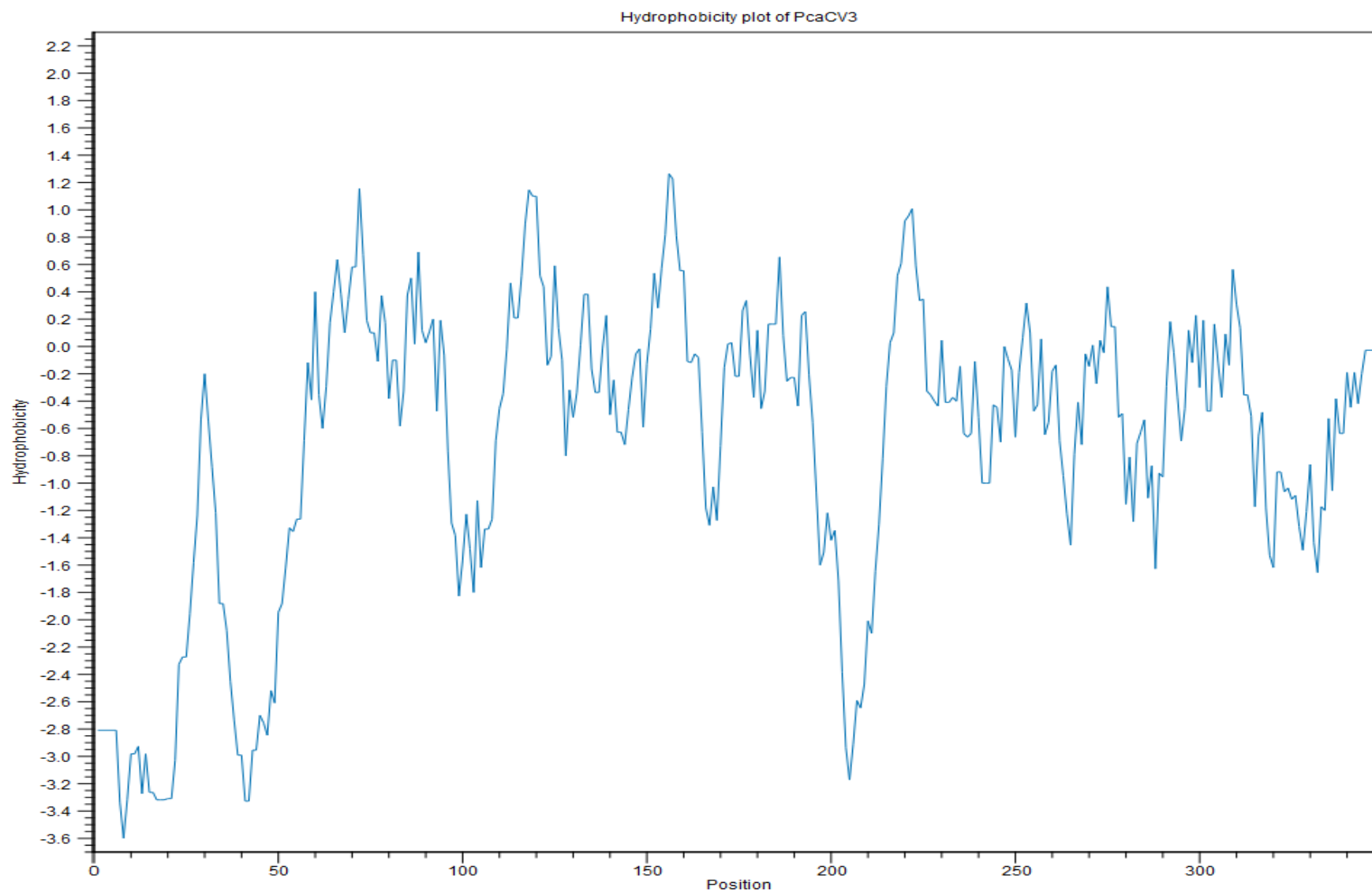

Supplement: S9 Fig — (PDF) [file pone.0166093.s009.pdf]

**S10 Fig. Hydrophobic plot of hypothetical capsid protein of PcaCV4.**

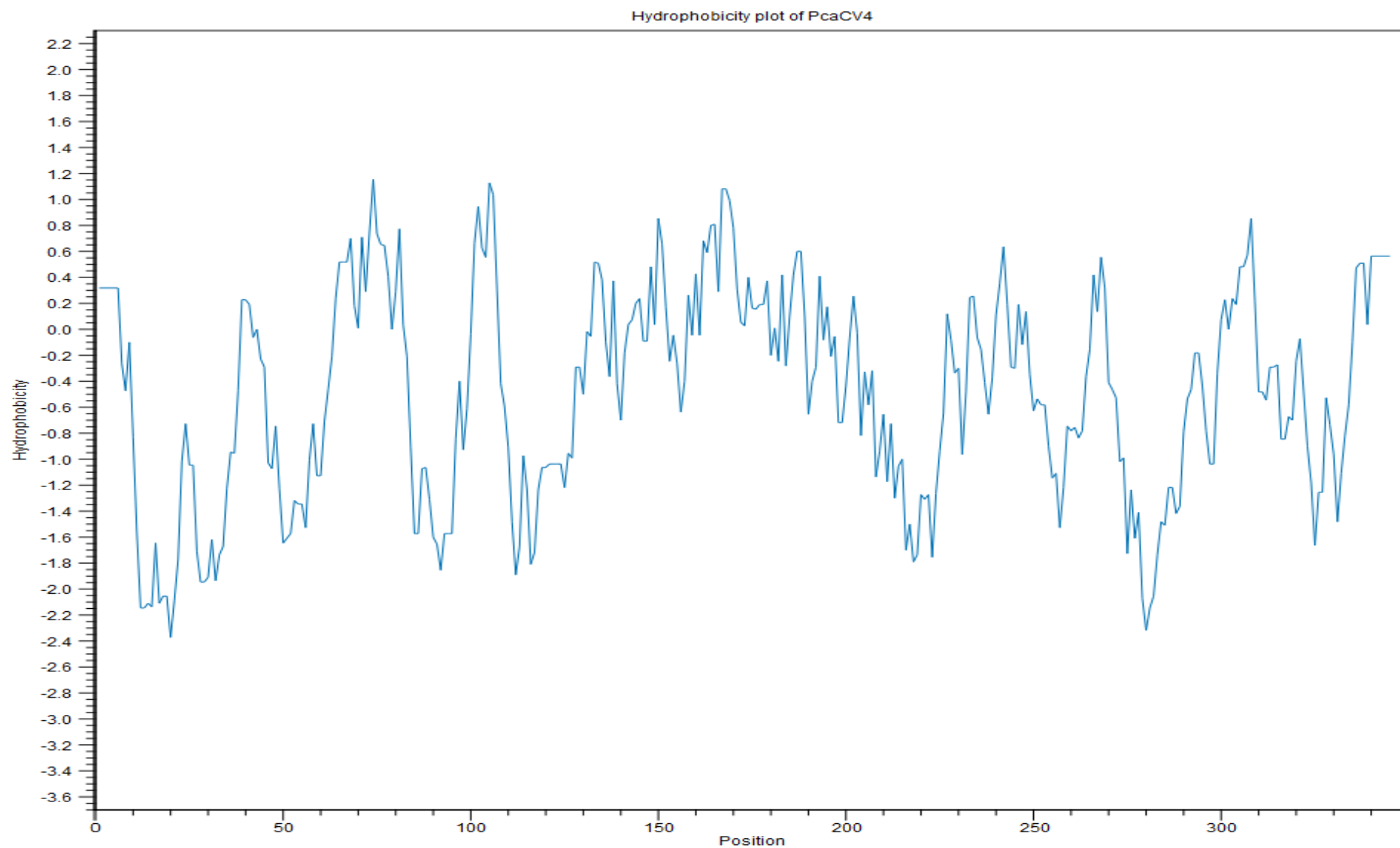

Supplement: S10 Fig — (PDF) [file pone.0166093.s010.pdf]
