# Supplementary material for: Novel Circular Single-Stranded DNA Viruses among an Asteroid, Echinoid and Holothurian (Phylum: Echinodermata)
Source: PLoS One. 2016 Nov 17;11(11):e0166093. doi: 10.1371/journal.pone.0166093 (PMC5113903; doi:10.1371/journal.pone.0166093)
Supplement: S1 Table — Conserved amino acid motifs obtained from alignments of putative Rep gene using MUCLE. (PDF) [file pone.0166093.s011.pdf]

S1 Table. **RCR and SF3 Helicase motifs found in circular ssDNA viruses. Conserved amino acid motifs obtained from alignments of putative *Rep* gene using MUCLE.**

	RCR Motifs			SF3 Helicase Motifs		
	I	II	III	Walker A	Walker B	Walker C
Circovirus	[CV]FT[LI]NN	PHLQG	YC[Sx]K	GP[Ps][Gc]xGKS	[VI][IML]DDF	UTSN
Cyclovirus	[CV]FT[WL]NN	[Px]HLQG	YC[Sx]K	G[Px][Pt]GxGKS	[IV][IU]DDF	UTS[Ne]
Geminivirus	FLTY[Ps]x	[Px]H[Lx]H[VAC]	Y[UAC]xK	Gx[ST]R[TI]GK[TS]	[VI][IV]DD[VI]	UL[Cx]N
Nanovirus	[VCx]FT[LI]N[FYN]	xHUQG	Y[CAS]xK	G[PS]xG[GN]EGK[TS]	[VIW][UAC][FIM]D[IVF]	V[FMI][AC]N
AfaCV2	MFTLFV	LHVQG	YCSK	GDPGSGKT	IFDDF	ITSN
AfaCV3	CFTDFK	KHNQG	YCSK	GKAGTGKT	LIDDF	ITSN
AfaCV4	CFTLNN	KHLQG	YACK	GPTGSGKT	LMDDF	ITSN
AfaCV5	VFTNYD	PHHQG	YCSK	GPSGAGKS	ILNEF	ITSV
SdaCV1	CFTLNN	PHLQG	YCSK	--------	----	----
SdaCV2	CWTLNN	PHLQG	YCKK	GPTGTGKT	LFDDF	ITSN
PcaCV1	VFTLNN	PHYQG	YCEK	GDTGVGKS	IINDF	ITSS
PcaCV2	VYTWNN	EHFQG	YSSK	GEPGTGKT	LLDDF	ITTN
PcaCV3	CFTWFA	PHVQG	----	GPTGVGKT	ILDDF	ITSN
PcaCV4	CLTINN	KHLQV	YCSK	GVTGSGKT	IIDDM	ITTT

U = I, L, V, M, F, Y, W
