# Supplementary material for: Novel Circular Single-Stranded DNA Viruses among an Asteroid, Echinoid and Holothurian (Phylum: Echinodermata)
Source: PLoS One. 2016 Nov 17;11(11):e0166093. doi: 10.1371/journal.pone.0166093 (PMC5113903; doi:10.1371/journal.pone.0166093)
Supplement: S2 Table — (PDF) [file pone.0166093.s012.pdf]

**S2 Table. *Rep* sequences pulled from NCBI for SDT analysis.**

| | |
|---|---|
| GoCV | gi\|76009487\|gb\|ABA39137.1\| Rep [Goose circovirus] |
| RW-A | gi\|254688518\|ref\|YP_003084282.1\| putative Rep protein [Circovirus-like genome RW-A] |
| RW-C | gi\|254688525\|ref\|YP_003084287.1\| putative Rep protein [Circovirus-like genome RW-C] |
| BatCV09 | gi\|342356290\|gb\|AEL28803.1\| replication-associated protein, partial [Bat circovirus ZS/Yunnan-China/2009] |
| SOG00160 | gi\|429890862\|gb\|AGA18245.1\| hypothetical protein [uncultured marine virus] |
| SOG03994 | gi\|429890944\|gb\|AGA18263.1\| hypothetical protein [uncultured marine virus] |
| GOM00583 | gi\|429891106\|gb\|AGA18314.1\| hypothetical protein [uncultured marine virus] |
| GOM02856 | gi\|429891193\|gb\|AGA18339.1\| hypothetical protein [uncultured marine virus] |
| MarineCRESS1 | gi\|429891313\|gb\|AGA18377.1\| hypothetical protein [uncultured marine virus] |
| SI00142 | gi\|429891345\|gb\|AGA18387.1\| hypothetical protein [uncultured marine virus] |
| MarineCRESS2 | gi\|429891360\|gb\|AGA18391.1\| hypothetical protein [uncultured marine virus] |
| SI00850 | gi\|429891424\|gb\|AGA18409.1\| hypothetical protein [uncultured marine virus] |
| SI03654 | gi\|429891524\|gb\|AGA18434.1\| hypothetical protein [uncultured marine virus] |
| MarineCRESS3 | gi\|444298002\|dbj\|GAC77838.1\| replication protein [uncultured marine virus] |
| MarineCRESS4 | gi\|444298006\|dbj\|GAC77836.1\| replication protein, partial [uncultured marine virus] |
| MarineCRESS5 | gi\|444298044\|dbj\|GAC77817.1\| replication protein, partial [uncultured marine virus] |
| MarineCRESS6 | gi\|444298094\|dbj\|GAC77792.1\| replication protein, partial [uncultured marine virus] |
| MarineCRESS7 | gi\|444298146\|dbj\|GAC77766.1\| replication protein, partial [uncultured marine virus] |
| LM28925 | gi\|459116743\|gb\|AGG39829.1\| replication-associated protein [Diporeia sp. associated circular virus] |
| PGV | gi\|508182168\|ref\|YP_008052687.1\| Rep domain protein [Phaeocystis globosa virus] |
| FdaCV | gi\|528890055\|gb\|AGS47835.1\| replication-associated protein [Farfantepenaeus duorarum circovirus] |
| SaCV-12 | gi\|664651384\|gb\|AIF34802.1\| replication-associated protein [Sewage-associated circular DNA virus-12] |
| BatCV | gi\|665518995\|gb\|AIF76250.1\| Rep [Bat circovirus] |
| LDMD15 | gi\|725829244\|ref\|YP_009109675.1\| replication-associated protein [Circoviridae 15 LDMD-2013] |
| LDMD21 | gi\|725829260\|ref\|YP_009109686.1\| replication-associated protein [Circoviridae 21 LDMD-2013] |
| AHEaCV2 | gi\|761263102\|gb\|AJP36339.1\| replication-associated protein, partial [Avon-Heathcote Estuary associated circular virus 2] |

| | |
|---|---|
| AHEaCV6 | gi\|761263147\|gb\|AJP36362.1\| replication-associated protein [Avon-Heathcote Estuary associated circular virus 6] |
| AHEaCV5 | gi\|765702617\|ref\|YP_009126884.1\| replication-associated protein [Avon-Heathcote Estuary associated circular virus 5] |
| AHEaCV9 | gi\|765702634\|ref\|YP_009126896.1\| replication-associated protein [Avon-Heathcote Estuary associated circular virus 9] |
| AHEaCV19 | gi\|765702674\|ref\|YP_009126925.1\| replication-associated protein [Avon-Heathcote Estuary associated circular virus 19] |
| EquCy | gi\|850199434\|gb\|AKN50607.1\| Rep protein [Cyclovirus Equ1] |
| FCCV | gi\|922073305\|ref\|YP_009163918.1\| putative replication initiation protein [Fiddler Crab associated circular virus] |
| CalCV | gi\|922073318\|ref\|YP_009163927.1\| putative replication initiation protein [Calanoida sp. copepod associated circular virus] |
| DLaCV6 | gi\|927769377\|gb\|ALE29847.1\| replication associated protein, partial [Dragonfly larvae associated circular virus-6] |
