# Supplementary material for: Novel Circular Single-Stranded DNA Viruses among an Asteroid, Echinoid and Holothurian (Phylum: Echinodermata)
Source: PLoS One. 2016 Nov 17;11(11):e0166093. doi: 10.1371/journal.pone.0166093 (PMC5113903; doi:10.1371/journal.pone.0166093)
Supplement: S3 Table — (PDF) [file pone.0166093.s013.pdf]

S3 Table. GenBank accession #s for novel virus genomes recovered in this study.

EchinoCRESS.sqn	AfaCV3	KX246255
EchinoCRESS.sqn	AfaCV4	KX246256
EchinoCRESS.sqn	AfaCV2	KX246257
EchinoCRESS.sqn	AfaCV5	KX246258
EchinoCRESS.sqn	SdaCV2	KX246259
EchinoCRESS.sqn	SdaCV1	KX246260
EchinoCRESS.sqn	PcaCV1	KX246261
EchinoCRESS.sqn	PcaCV4	KX246262
EchinoCRESS.sqn	PcaCV3	KX246263
EchinoCRESS.sqn	PcaCV2	KX246264
